# Limits to sustained energy intake XXV: milk energy output and thermogenesis in Swiss mice lactating at thermoneutrality

**DOI:** 10.1038/srep31626

**Published:** 2016-08-24

**Authors:** Zhi-Jun Zhao, Li Li, Deng-Bao Yang, Qing-Sheng Chi, Catherine Hambly, John R. Speakman

**Affiliations:** 1 School of Life and Environmental Sciences, Wenzhou University, Wenzhou, Zhejiang 325027, China; 2 State Key Laboratory of Molecular Developmental Biology, Institute of Genetics and Developmental Biology, Chinese Academy of Sciences, Beijing 100100, China; 3 State Key Laboratory of Integrated Management for Pest Insects and Rodents, Institute of Zoology, Chinese Academy of Sciences, Beijing 100080, China; 4 Institute of Biological and Environmental Sciences, University of Aberdeen, Aberdeen, Scotland, UK

## Abstract

Previous studies at 21 °C and 5 °C suggest that in Swiss mice sustained energy intake (SusEI) and reproductive performance are constrained by the mammary capacity to produce milk. We aimed to establish if this constraint also applied at higher ambient temperature (30 °C). Female Swiss mice lactating at 30 °C had lower asymptotic food intake and weaned lighter litters than those at 21 °C. Resting metabolic rate, daily energy expenditure, milk energy output and suckling time were all lower at 30 °C. In a second experiment we gave mice at 30 °C either 6 or 9 pups to raise. Female performance was independent of litter size, indicating that it is probably not controlled by pup demands. In a third experiment we exposed only the mother, or only the offspring to the elevated temperature. In this case the performance of the mother was only reduced when she was exposed, and not when her pups were exposed, showing that the high temperature directly constrains female performance. These data suggest that at 30 °C SusEI and reproductive performance are likely constrained by the capacity of females to dissipate body heat, and not indirectly via pup demands. Constraints seem to change with ambient temperature in this strain of mouse.

Lactation is the time of greatest energy demand in most mammals^1^,^2^,^3^. The limits on sustained energy intake (SusEI) during peak lactation are important because they determine the total investment that mammals can make in their offspring and may therefore define maximum litter and offspring sizes^4^,^5^. Several hypotheses have been previously advanced to explain the limits on female lactation performance. The ‘peripheral limitation’ hypothesis suggests that the capacity of the mammary gland to secrete milk likely sets the limitation on SusEI^6^,^7^,^8^,^9^,^10^,^11^. In contrast, the ‘heat dissipation limit’ (HDL) idea suggests that females are constrained by the maximal capacity to dissipate body heat generated as a by-product of processing food and producing milk during peak lactation^12^,^13^,^14^.

Data from small mammals with respect to these ideas has been inconclusive (see also^13^,^14^,^15^). For example, MF1 mice and Brandt’s voles (*Lasiopodomys brandtii*) lactating in hot conditions (30 °C) had significantly lower mean asymptotic food intake and reproductive output than those at room (21 °C) or cold temperatures (8 °C), providing support for the HDL idea^12^,^16^,^17^. In contrast, Hammond *et al*.^9^ found that when half of the mammary glands were surgically removed, Swiss Webster mice did not respond by elevating either milk production or food intake^9^, suggesting that the mammary gland was indeed the point at which the system was peripherally limited. However, Swiss mouse females increase their peak intake when exposed to the cold^6^ and significantly increased milk energy output at the cold temperature (5 °C) compared with their counterparts lactating at warm conditions (23 °C) but only when raising small litters^18^.

Manipulating ambient temperature may have complex effects because it not only alters a females’ capacity to dissipate heat, but also directly affects the offspring^19^. Several studies have therefore used the shaved female as an experimental paradigm to test the heat dissipation limit idea, on the basis that shaving elevates the female’s capacity to dissipate heat, without directly affecting her pups. However, such studies have generated inconsistent results. In MF1 mice^20^ and bank voles^21^ shaving elevated milk production and pup growth. In short-tailed field voles shaving elevated pup growth but a small impact on milk production did not reach significance^19^. In contrast in both hamsters^2^ and Swiss mice^11^,^22^ shaving did not increase milk production, despite elevated asymptotic food intake.

To bring some clarity to these disparate results it was proposed that both peripheral and heat dissipation limits are probably important in all animals, but to different extents^15^. It was also suggested that animals may be limited at different levels and that may change as conditions vary^15^. For example, MF1 mice, probably have higher maximum milk production capacity relative to their capacity to dissipate body heat, resulting in a consistent limitation by their heat dissipation capacity. In consequence there is an inverse relationship between temperature and food intake/milk production across the range from 30 °C to 5 °C^12^,^23^. Contrasting this response, Swiss mice might have high capacity to dissipate body heat compared with their maximum milk production capacity. Hence females were not able to increase milk production when transferred into cold conditions^18^ or when they were dorsally shaved^11^,^22^ because their capacity to dissipate heat was elevated, but their mammary glands were unable to respond.

Because at 21 °C the performance of MF1 and Swiss mice is similar, a test of this idea would be to expose Swiss mice to high ambient temperatures during lactation. If the heat dissipation limits apply in this strain, but only at high temperatures, then we would anticipate SusEI and lactation performance would decline in the same way that performance declines in MF1 mice^12^. In contrast if the performance was unaltered in hot, compared to cooler conditions, then we would have evidence that the heat dissipation limits idea is refuted across the whole temperature range in this strain.

In the present study, we exposed female Swiss mice to a hot conditions (30 °C) during lactation and determined food intake, digestible energy intake, litter size, litter mass, daily energy expenditure (DEE) and milk energy output (MEO). We also measured brown adipose tissue (BAT) uncoupling protein 1 (UCP1) content and gene expression of carnitine palmitoyltransferase 1B (*cpt1b*) which are involved in thermogenesis and control of long-chain fatty acid oxidation^24^,^25^. Since ambient temperature effects on lactation of the females may be mediated via effects on the pups, we performed two additional experiments that aimed to separate if lactation performance at 30 °C was driven by pup demands, or by factors intrinsic to the female. These involved experimentally varying the litter size, and exposing either the female alone or the pups alone to the elevated temperature (see refs 26,27 for similar approach).

## Results

### Experiment 1

#### Body mass

**Figure 1:**
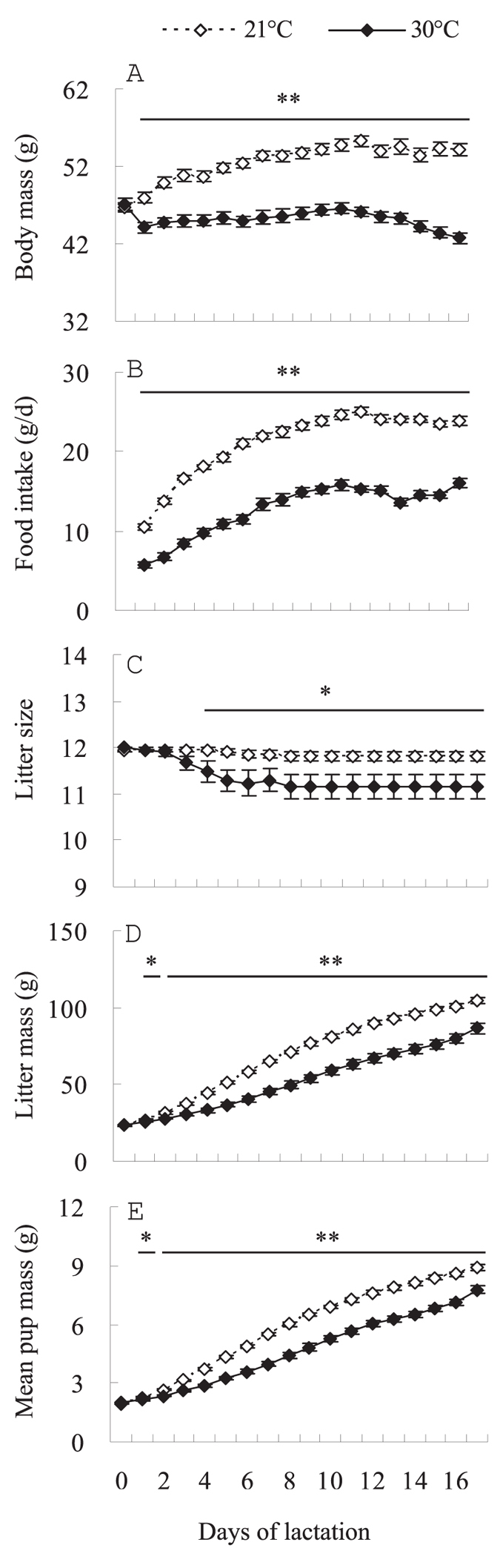
Body mass (**A**), food intake (**B**), litter size (**C**), litter mass (**D**) and mean pup mass (**E**) during lactation in Swiss mice at 21 and 30 °C. Females were allowed to raise 12 pups only from the day of parturition (day 0) and were exposed to either 21 (*n* = 21) or 30 °C (*n* = 21) from day 1 of lactation. Data are means ± s.e.m. *Significant difference between the two groups (*P* < 0.05), **P < 0.01.

Body mass was not different between the two groups on the day of parturition (by convention day 0 of lactation^4^): (*t*_40_ = 0.4, *P* > 0.05, Fig. 1A). In females lactating at 21 °C, body mass increased significantly throughout lactation, (day 0–17, *F*_17, 340_ = 59.2, *P* < 0.01) and was increased by 16% on day 17 compared to day 0. In contrast, females lactating at 30 °C significantly decreased their weight during lactation (day 0–17, *F*_17, 340_ = 10.3, *P* < 0.01) and on day 17 body mass had decreased by 9% relative to that on day 0. A significant difference in body mass was observed between the two groups throughout lactation, with females at 30 °C lighter than those at 21 °C on day 1 and thereafter (day 1, *t*_40_ = 4.2, *P* < 0.01). On the weaning day (17), body mass was 21% lower at 30 °C than 21 °C (day 17, *t*_40_ = 10.3, *P* < 0.01, Fig. 1A).

#### Food intake

Food intake averaged 10.5 ± 0.4 g/d and 5.7 ± 0.4 g/d at 21 °C and 30 °C on day 1 (day 1, *t*_40_ = 9.0, *P* < 0.01, Fig. 1B). Food intake increased significantly throughout lactation at 21 °C and also at 30 °C. On average food intake increased by 126% and 183% at 21 °C and 30 °C, respectively, on day 17 compared with that on day 1 (21 °C, *F*_16, 320_ = 147.6, *P* < 0.01; 30 °C, *F*_16, 320_ = 77.8, *P* < 0.01). Asymptotic food intake was 24.2 ± 0.3 g/d and 15.0 ± 0.4 g/d at 21 °C and 30 °C, respectively; females lactating at 30 °C had 38.0% lower asymptotic food intake than those lactating at 21 °C (*t*_40_ = 17.7, *P* < 0.01, Fig. 1B).

#### Litter size

Before litters were manipulated on day 0, litter size averaged 11.4 ± 0.5 and 11.4 ± 0.5 in 21 °C and 30 °C groups, respectively (*t*_40_ = 0.1, *P* > 0.05, Fig. 1C). On day 5 and thereafter, females had fewer offspring at 30 °C than at 21 °C (day 5, *t*_40_ = 2.6, *P* < 0.05). On day 17, litter size averaged 11.8 ± 0.1 and 11.1 ± 0.3 in 21 °C and 30 °C groups, respectively, and females lactating at 30 °C weaned significantly smaller litters (by 5.9%) than those lactating at 21 °C (day 17, *t*_40_ = 2.4, *P* < 0.05, Fig. 1C).

#### Litter mass and mean pup mass

**Figure 2:**
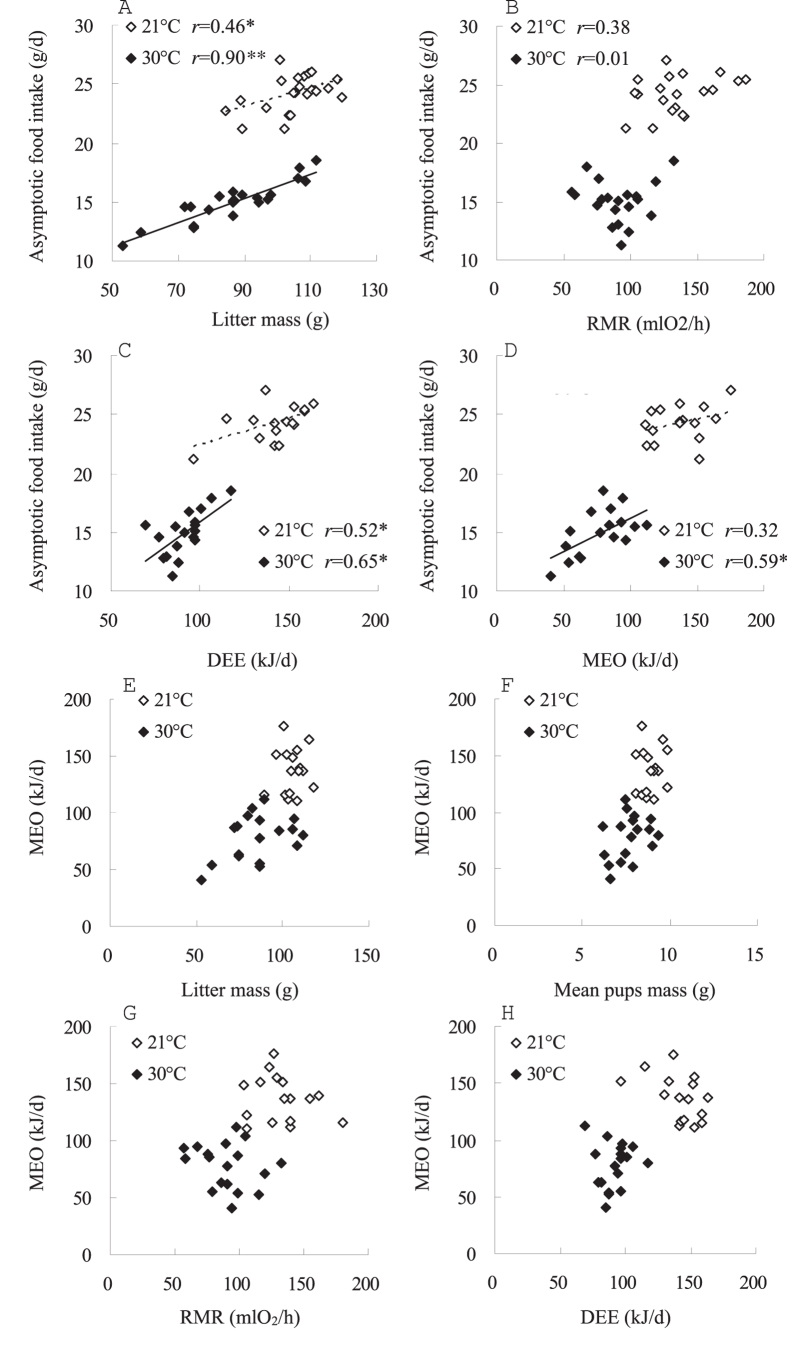
Correlations between asymptotic food intake and liter mass (**A**), RMR (**B**), DEE (**C**) and MEO (**D**), as well as between MEO and liter mass (**E**), mean pup mass (**F**), RMR (**G**) and DEE (**H**) in Swiss mice lactating at 21 (*n* = 21) and 30 °C (*n* = 21).

Litter mass was not different between the two groups before litters were manipulated on day 0 (*t*_40_ = 0.4, *P* > 0.05, Fig. 1D). Litter mass increased significantly throughout lactation in both 21 °C and 30 °C groups. Litter mass was increased by 3.5 times on day 17 relative to day 0 at 21 °C (*F*_17, 340_ = 1724.1, *P* < 0.01) but increased by only 2.7 times at 30 °C (*F*_17, 340_ = 338.4, *P* < 0.01). On day 1 and thereafter, litter mass was significantly lower at 30 °C than 21 °C (day 1, *t*_40_ = 2.2, *P* < 0.05). On day 17, litters were weaned weighing 104.8 ± 2.0 g and 86.7 ± 3.4 g in the 21 and 30 °C groups, respectively, which was equivalent to 17% lower at 30 °C (day 17, *t*_40_ = 4.6, *P* < 0.01, Fig. 1D). Litter mass was positively correlated with asymptotic food intake at 21 and 30 °C (21 °C, *r* = 0.46, *P* < 0.05, 30 °C, *r* = 0.90, *P* < 0.01, Fig. 2A).

Mean pup mass averaged 1.97 ± 0.03 g and 1.98 ± 0.03 g on day 0 in 21 and 30 °C groups, respectively, and no difference was observed between the two groups at this age (*t*_40_ = 0.2, *P* > 0.05, Fig. 1E). On day 1 and thereafter, mean pup mass was lower at 30 °C than at 21 °C (*t*_40_ = 2.3, *P* < 0.05). On day 17, pups were weaned with an average weight of 8.87 ± 0.16 g at 21 °C and 7.76 ± 0.21 g at 30 °C, and mean pup mass was lower by 12.6% at 30 °C than at 21 °C (*t*_40_ = 4.3, *P* < 0.01, Fig. 1E).

#### Suckling times

**Figure 3:**
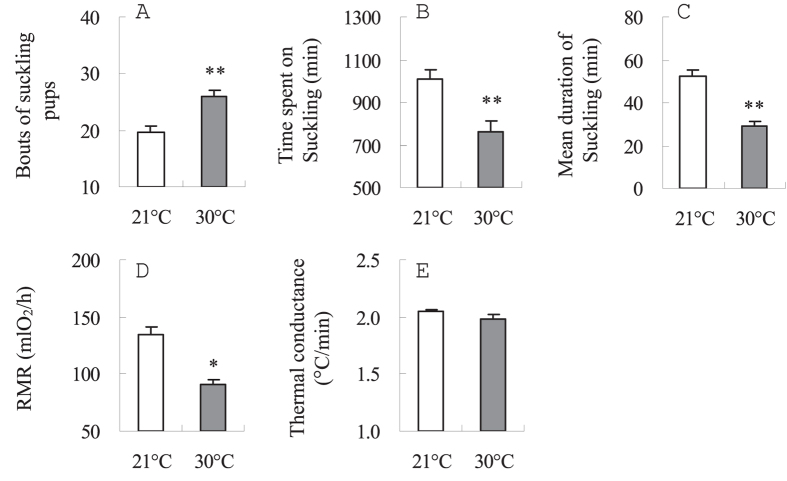
Bouts of sucking pups (**A**), time spent on suckling pups (**B**), mean duration of suckling (**C**), RMR (**D**) and thermal conductance (**E**) in Swiss mice lactating at 21°C (*n* = 8) and 30°C (*n* = 8). Data are means ± s.e.m. *Significant difference between the two groups (P < 0.05), **P < 0.01.

The number of suckling bouts was significantly different between the two groups, with mothers at 30 °C having 31.6% more bouts of suckling pups than mothers at 21 °C (*t*_14_ = 4.4, *P* < 0.01, Fig. 3A). During peak lactation, females lactating at 30 °C spent 25.0% less time on suckling pups than females lactating at 21 °C (*t*_14_ = 3.7, *P* < 0.01, Fig. 3B). Mean duration of suckling was also lower in females at 30 °C than at 21 °C (*t*_14_ = 6.1, *P* < 0.01, Fig. 3C).

#### Resting metabolic rate (RMR) and thermal conductance

RMR averaged 135.2 ± 5.9 and 91.0 ± 4.6 mlO_2_/h in 21 and 30 °C groups, respectively. Females lactating at 30 °C had 32.7% lower RMR than females lactating at 21 °C (*F*_1, 35_ = 5.5, *P* < 0.05, Fig. 3D). Body mass was on average 50.7 ± 0.9 and 41.0 ± 0.8 g in 21 and 30 °C groups, respectively, and the effect of body mass on RMR was not statistically significant (*F*_1, 35_ = 1.9, *P* > 0.05). No difference in thermal conductance of fur was observed between 21 and 30 °C (*t*_18_ = 1.36, *P* > 0.05, Fig. 3E). No correlations were observed between RMR and asymptotic food intake at either 21 or 30 °C (21 °C, *r* = 0.38, *P* > 0.05, 30 °C, *r* = 0.01, *P* > 0.05, Fig. 2B).

#### Energy intake, DEE and MEO

Females at 30 °C had significantly lower GEI and DEI than females at 21 °C (Table 1). MEI of females at 30 °C was 40.1% lower than that at 21 °C, while digestibility did not differ between the two groups. DEE and MEO were also significantly different between the two groups, which were 35.4% and 43.2% lower at 30 °C than at 21 °C (Table 1). DEE was positively correlated with asymptotic food intake at both 21 and 30 °C (21 °C, *r* = 0.52, *P* < 0.05, 30 °C, *r* = 0.65, *P* < 0.05, Fig. 2C). There was a significant correlation between MEO and asymptotic food intake at 30 °C (*r* = 0.59, *P* < 0.05), while the correlation was not statistically significant at 21 °C (*r* = 0.32, *P* > 0.05, Fig. 2D). No correlations were observed between MEO and litter mass (Fig. 2E), mean pup mass (Fig. 2F), RMR (Fig. 2G) and DEE (Fig. 2H)(*P* > 0.05).

#### Body composition

Both wet and dry carcass masses were significant higher in females lactating at 21 °C than at 30 °C (Table 2). Wet and dry masses of liver, heart, spleen and kidneys were also higher at 21 °C than at 30 °C. The gastrointestinal tracts including stomach, small and large intestine and caecum were significantly heavier in females lactating at 21 °C than at 30 °C. No differences were observed in masses of brain and lung between the two groups (Table 2).

#### BAT cpt1b, pgc1α and ucp1 gene expression and UCP1 protein content

Neither BAT *cpt1b* nor *pgc1α* gene expression was affected by temperature (*cpt1b*, *t*_17_ = 0.20, *P* > 0.05; *pgc1α*, *t*_17_ = 0.14, *P* > 0.05, Supplementary materials, Fig. S1A). BAT *ucp1* gene expression and UCP1 protein content did not differ between females lactating at 21 and 30 °C (*ucp1, t*_17_ = 0.19, *P* > 0.05; UCP1, *t*_18_ = 0.44, *P* > 0.05, Fig. S1B).

### Experiment 2

**Figure 4:**
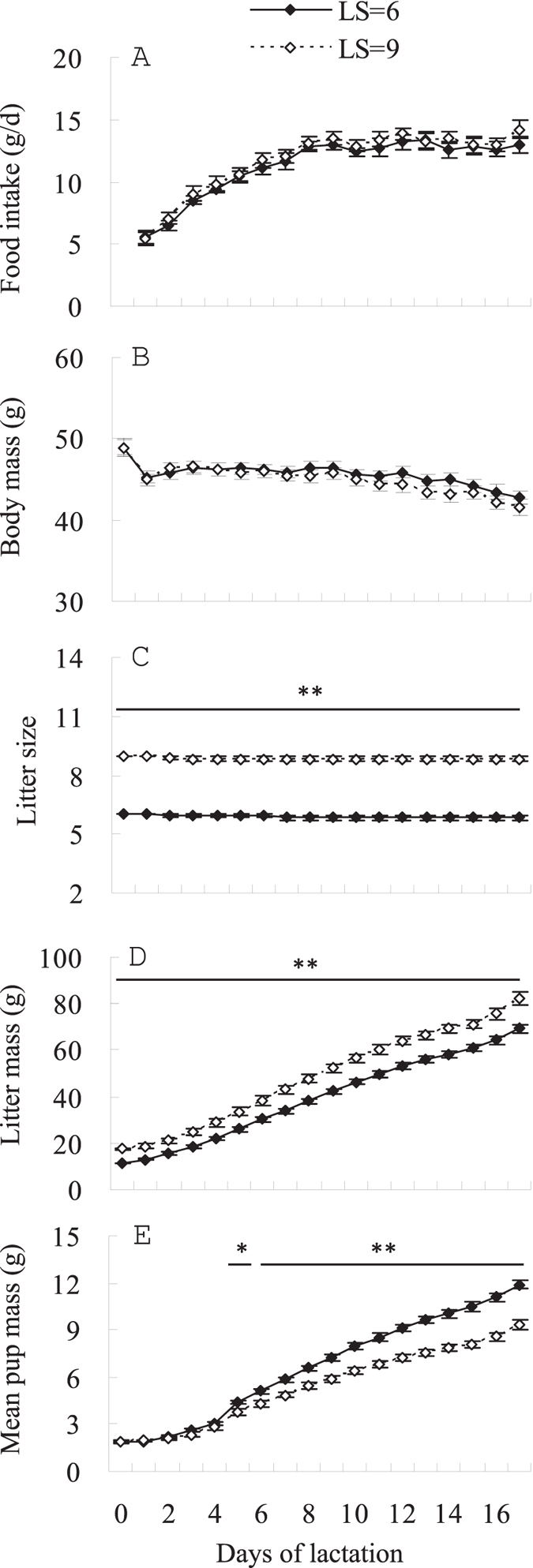
Food intake (**A**) and body mass (**B**), litter size (**C**), litter mass (**D**) and mean pup mass (**E**) of female Swiss mice raising 9 (LS = 9, *n* = 12) or 6 (LS = 9, *n* = 12) litters at 30 °C throughout lactation. Data are means ± s.e.m. *Significant difference between the two groups (*P* < 0.05), **P < 0.01.

*Food intake and body mass of females in LS* = *6 and LS* = *9 groups*. No difference was observed in food intake between females raising 6 and 9 litters on any day over lactation (e.g. day 1, *t*_22_ = 0.23, *P* > 0.05; day 17, *t*_22_ = 1.24, *P* > 0.05; Fig. 4A). Body mass decreased significantly throughout lactation in both groups, by 15.1% in the LS = 9 group (*F*_17, 187_ = 17.93, *P* < 0.01) and by 12.6% in the LS = 6 group (*F*_17, 187_ = 15.61, *P* < 0.01, Fig. 4B). The difference in body mass between the two groups was not significant on any day over the entire lactation (e.g. day 1, *t*_22_ = 0.02, *P* > 0.05; day 17, *t*_22_ = 0.95, *P* > 0.05).

#### Litter size, litter mass and mean pups mass of females in LS = 6 and LS = 9 groups

Mean litter size was 8.8 ± 0.1 and 5.8 ± 0.1 litters in LS = 9 and LS = 6 groups, respectively (day 17, *t*_22_ = 18.88, *P* < 0.01; Fig. 4C). Litter mass averaged 17.5 ± 0.5 g and 11.5 ± 0.2 g in LS = 9 and LS = 6 groups, respectively, after the litter size manipulation on parturition day (day 0, *t*_22_ = 10.70, *P* < 0.01) and weaned 82.2 ± 2.9 g and 69.3 ± 1.8 g (day 17, *t*_22_ = 3.77, *P* < 0.01, Fig. 4D). Pups weight did not differ between the two groups following the manipulation on the day of parturition (day 0, *t*_22_ = 0.30, *P* > 0.05; Fig. 4E). Litters in the LS = 6 group gained more weight than those in the LS = 9 group and a significant difference was observed on day 5 and thereafter (day 5, *t*_22_ = 2.56, *P* < 0.05). On the day of weaning, mean pup mass was 11.9 ± 0.2 g in LS = 6 group, which was higher by 27.5% than 9.3 ± 0.4 g of pups in LS = 9 group (*t*_22_ = 5.97, *P* < 0.01).

#### RMR, GEI, DEE and MEO

Neither RMR of females nor litters differed between LS = 9 and LS = 6 groups (Table 3). No differences in GEI, DEI and MEI were observed between the two groups (Table 3). There were also no differences in DEE and MEO between LS = 9 and LS = 6 groups (Table 3).

### Experiment 3

#### Body mass

**Figure 5:**
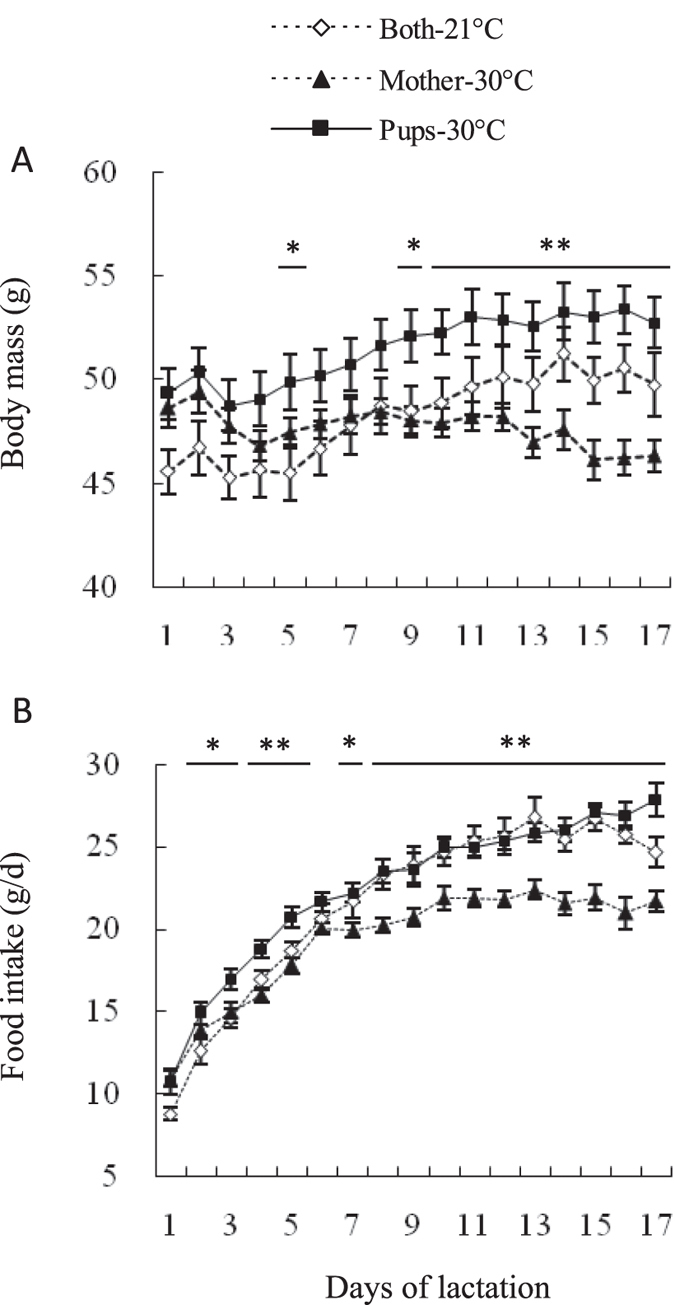
Body mass (**A**) and food intake (**B**) in lactating Swiss mice that were consecutively stopped from suckling pups in an interval of 90 min on day 1 till 17. Both-21 °C (*n* = 11), both mothers and pups were maintained at 21 °C when mothers were moved out; Mother-30 °C (*n* = 15), mothers were moved out to 30 °C and pups were still maintained at 21 °C; Pups-30 °C (*n* = 15), mothers were moved out but still maintained at 21 °C, while home cage with pups were exposed to 30 °C. Both mothers and pups were maintained at 21 °C in three groups when mothers were back in the home cage. *Difference among the three groups were significant (*P* < 0.05), **P < 0.01. Data are means ± s.e.m.

Body mass did not differ among the three groups on day 1 of lactation (*F*_2,38_ = 2.92, *P* > 0.05, Fig. 5A). Significant group differences were observed on day 5, day 9 and thereafter (d 5, *F*_2,38_ = 3.38, *P* < 0.05; d 9, *F*_2,38_ = 4.54, *P* < 0.05, d 17, *F*_2,38_ = 8.36, *P* < 0.01). In the ‘Mother-30 °C’ group, body mass decreased significantly throughout lactation (d 1–17, *F*_16, 224_ = 3.62, *P* < 0.01), equal to a loss of 4.7% on day 17 compared with that on day 1. Conversely females in ‘Both-21 °C’ and ‘Pups-30 °C’ groups gained weight during lactation (d 1–17, Both-21 °C, *F*_16, 160_ = 22.88, *P* < 0.01; Pups-30 °C, *F*_16, 224_ = 20.82, *P* < 0.01, Fig. 5A) equal to 9.1% and 6.9% respectively on day 17 relative to that on day 1.

#### Food intake

On day 1, food intake averaged 8.8 ± 0.4, 10.9 ± 0.8 and 10.8 ± 0.8 g/d in ‘Both-21 °C’, ‘Mother-30 °C’ and ‘Pups-30 °C’ (*F*_2,35_ = 3.24, *P* > 0.05, Fig. 5B). From day 2 until 17 of lactation, with the exception of day 6, food intake was significantly different between the three groups (e.g. d 2, *F*_2,35_ = 3.54, *P* < 0.05, d17, *F*_2,35_ = 13.76, *P* < 0.01). Food intake in ‘Both-21 °C’ and ‘Pups-30 °C’ significantly increased throughout lactation (d 1–17, Both-21 °C, *F*_16, 144_ = 110.03, *P* < 0.01; Pups-30 °C, *F*_16, 208_ = 82.14, *P* < 0.01), equivalent to 1.8 and 1.6 fold on day 17 compared with that on day 1. In the ‘Mother-30 °C’ group however, food intake increased significantly from day 1 to day 7 (d 1-d7, *F*_6, 78_ = 68.30, *P* < 0.01), and then did not increase any more, but reached a plateau (d 8–17, *F*_9, 117_ = 1.70, *P* > 0.05). Asymptotic food intake averaged 25.8 ± 0.7, 21.7 ± 0.5 and 26.3 ± 0.6 g/d in ‘Both-21 °C’, ‘Mother-30 °C’ and ‘Pups-30 °C’ respectively. The food intake of the ‘Mother-30 °C’ group was 15.6% and 17.3% lower than other two groups (*F*_2,35_ = 20.68, *P* < 0.01).

**Figure 6:**
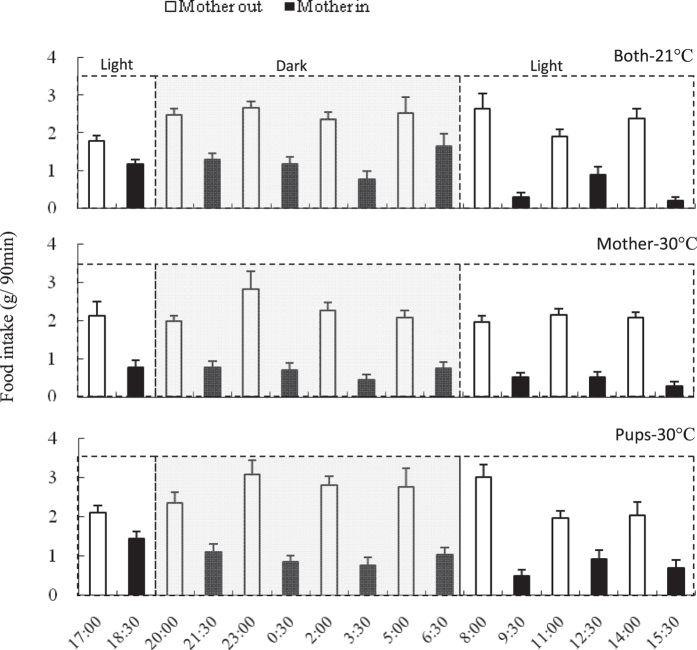
Food intake (g/90 min) of females when mother was moved out and back in home cage on a representative day (day 14) of lactation. Data are means ± s.e.m.

**Figure 7:**
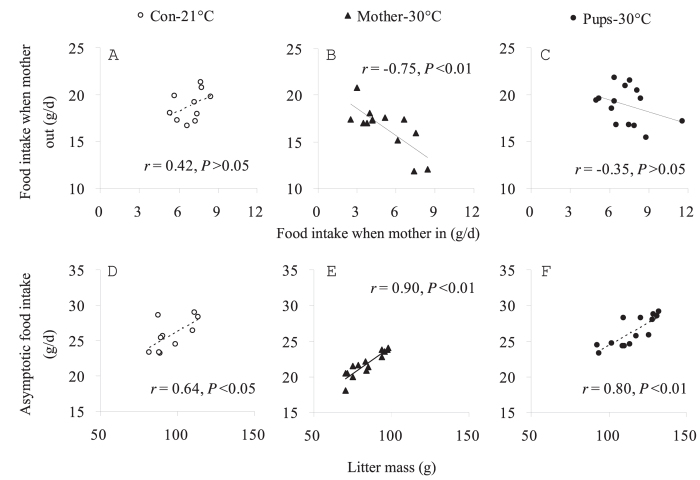
Correlations between food intake of females when suckling their pups in home cage and of females when being moved out (**A**–**C**) and correlation between asymptotic food intake and litter mass (**D**–**F**) in lactating Swiss mice.

The accumulated food intake between days 11 to 17 of lactation was not significantly different between the three groups when mother was moved out of the home cage (Table 4). However, when the mothers were back in the home cage and allowed to suckle their pups, food intake was significantly lower in the Mother-30 °C group than in other two groups from day 14 onwards. Hence the accumulated food intake on days 11 to 17 was lower by 28.8% and 32.7% in the ‘Mother-30 °C’ group than that in the ‘Both-21 °C’ and ‘Pups-30 °C’ groups, respectively (Table 4). In all three groups females consumed significantly more food when they were moved out of the home cage, than when they were in it (Fig. 6). There was no significant correlation between the food intake of the individuals during the time they spent in the home cage and that when in the other cage in ‘Both-21 °C’ and ‘Pups-30 °C’ groups, while a significant negative correlation was observed in the ‘Mother-30 °C’ group (Fig. 7A–C).

#### Litter size, litter mass and mean pup mass

**Figure 8:**
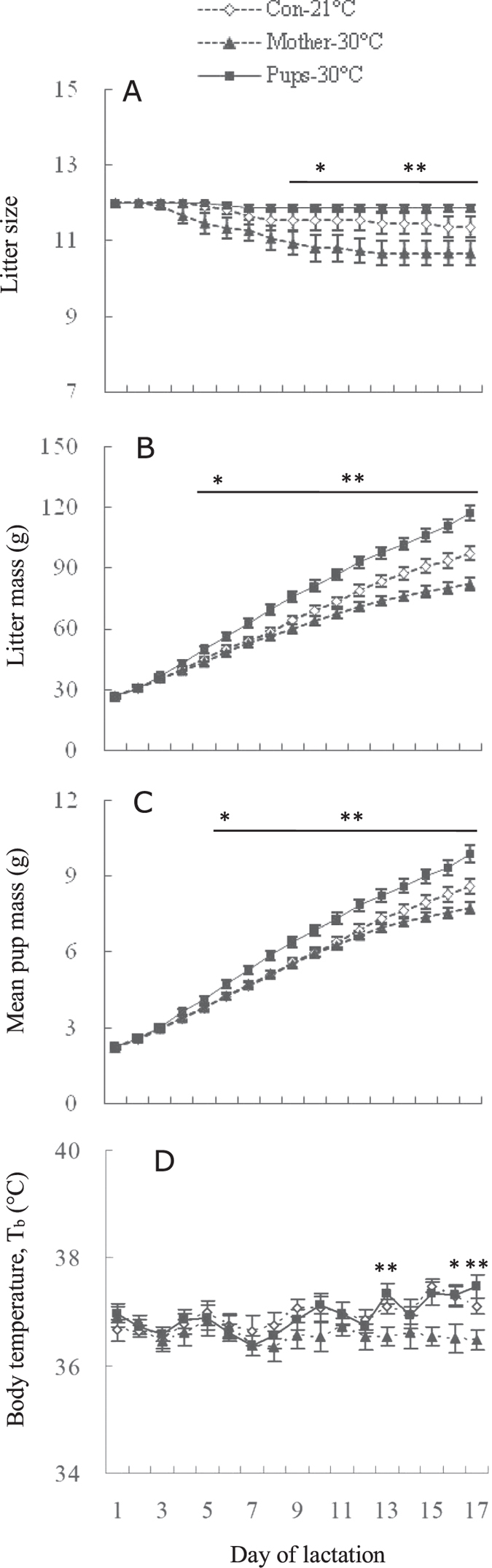
Litter size (**A**), litter mass (**B**), mean pup mass (**C**) and body temperature (**D**) in lactating Swiss mice that were consecutively stopped from suckling pups in an interval of 90 min on day 1 till 17. *Difference among the three groups were significant (*P* < 0.05), **P < 0.01. Data are means ± s.e.m. The three groups were defined as that in Fig. 5.

Litter size was regulated to 12 for each mother in three groups on day 1. Litter size in Mother-30 °C group decreased from 12.0 on day 1 to 10.7 on day 12 (d 1–12, *F*_11, 154_ = 1.96, *P* < 0.05), and was constant at 10.7 after day 12 of lactation (Fig. 8A). A significant group effect was observed on day 9 of lactation and afterwards. Females in the ‘Mother-30 °C’ group raised smaller litters than the other two groups (d 9, *F*_2, 38_ = 4.06, *P* < 0.05, post hoc, *P* < 0.05; d17, *F*_2, 38_ = 6.54, *P* < 0.01, post hoc, *P* < 0.05).

Litter mass was not different among three groups on day 1 (*F*_2, 38_ = 0.11, *P* > 0.05, Fig. 8B). A significant group difference was observed on day 5, when litter mass in ‘Mother-30 °C’ was lower than that in ‘Pups-30 °C’ group (*F*_2, 38_ = 4.08, *P* < 0.05, post hoc, *P* < 0.05). Litters were weaned at 97.3 ± 3.6 g, 82.3 ± 3.0 g and 117.3 ± 3.7 g in ‘Both-21 °C’, ‘Mother-30 °C’ and ‘Pups-30 °C’ groups, respectively (*F*_2,35_ = 27.89, *P* < 0.01). Litter mass in the ‘Mother-30 °C’ group was lower by 15.5% and 29.8% than that in the ‘Both-21 °C’, and the ‘Pups-30 °C’ group, respectively (post hoc, *P* < 0.05), and that of ‘Pups-30 °C’ group was significantly greater than the ‘Both-21 °C’ group (post hoc, *P* < 0.05). Litter mass was positively correlated with asymptotic food intake (Fig. 7D–F).

Mean pup mass did not differ among the three groups on day 1 (F_2, 38_ = 0.10, P > 0.05), but did on day 6 of lactation and afterwards (*F*_2, 38_ = 3.44, *P* < 0.05, Fig. 8C). On the day of weaning, mean pup mass was 8.6 ± 0.3 g, 7.7 ± 0.2 g and 9.9 ± 0.3 g in ‘Both-21 °C’, ‘Mother-30 °C’ and ‘Pups-30 °C’ groups respectively (*F*_2,35_ = 14.82, *P* < 0.01), which was lower by 10.0% and 21.8% in Mother-30 °C than that in Both-21 °C and Pups-30 °C groups, respectively (post hoc, *P* < 0.05). The mean pup mass of the ‘Pups-30 °C’ group was significantly greater than the ‘Both-21 °C’ group (post hoc, *P* < 0.05).

#### Body temperature (T_b_)

T_b_ was not different among the three groups on day 1 of lactation (*F*_2, 38_ = 0.68, *P* > 0.05, Fig. 8D). Significant differences in T_b_ between the three groups were observed on day 13, 16 and 17 (d13, *F*_2, 38_ = 5.93, *P* < 0.01, d 16, *F*_2, 38_ = 4.32, *P* < 0.05, d17, *F*_2, 38_ = 7.04, *P* < 0.01), and T_b_ in Mother-30 °C group was significant lower than that in Pups-30 °C group (post hoc, *P* < 0.05).

#### GEI, digestibility, DEE and MEO

**Figure 9:**
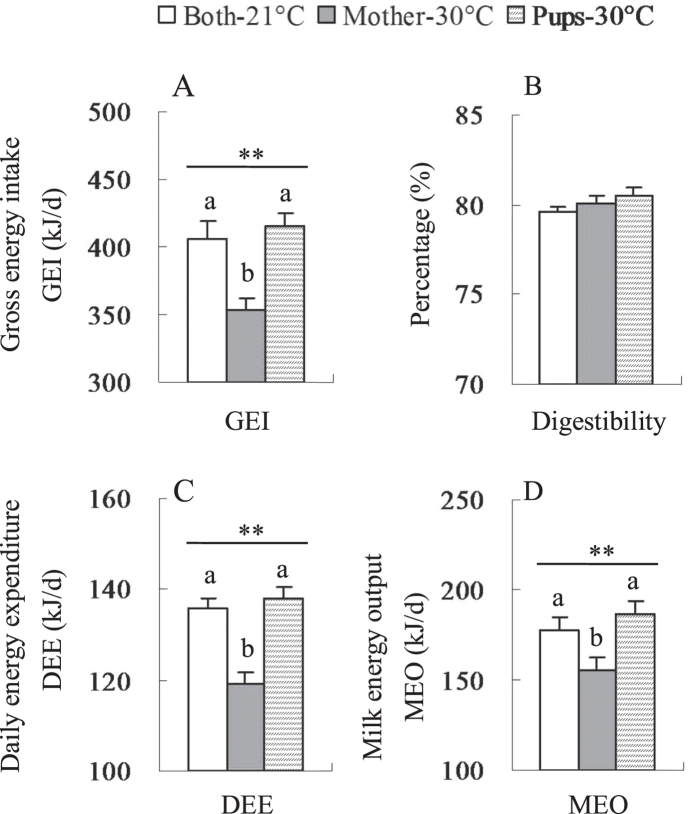
Gross energy intake (GEI, (**A**), digestibility (**B**), daily energy expenditure (DEE, (**C**) and milk energy output (MEO, (**D**) in lactating Swiss mice that were consecutively stopped from suckling pups in an interval of 90** **min on day 1 till 17. Data are means ± s.e.m. **Effect of temperatures was significant (*P* < 0.01). Different letters above the columns indicate significant difference between the three groups (*P* < 0.05). The three groups were defined as that in Fig. 5.

GEI was significantly different among the three groups (*F*_2, 31_ = 12.07, *P* < 0.01), and GEI in Mother-30 °C was lower by 13.1% and 15.0% than that in Both-21 °C and Pups-30 °C groups (post hoc, *P* < 0.05, Fig. 9A). There was no difference in digestibility among the three groups (*F*_2, 31_ = 1.01, *P* > 0.05, Fig. 9B). DEE was significantly lower in Mother-30 °C group than that in Both-21 °C and Pups-30 °C groups (*F*_2, 31_ = 15.97, *P* < 0.01, Fig. 9C). A significant difference in MEO was observed among the three groups, and females in ‘Mother-30 °C’ group showed 12.6% and 16.9% lower MEO than mothers in ‘Both-21 °C’ and ‘Pups-30 °C’ groups, respectively (*F*_2, 31_ = 5.78, *P* < 0.01, Fig. 9D). A significant positive correlation was observed between GEI and body mass in the ‘Both-21 °C’ group, but not in the ‘Mother-30 °C’ and ‘Pups-30 °C’ groups (Fig. S2A). GEI was positively correlated with DEE and MEO in all three groups (Fig. S2C,D). There were also significantly positive correlations between GEI and litter mass in the three groups (Fig. S3A).

#### RMR of females and litters

Females in the ‘Both-21 °C’ group did not have a significantly different RMR from females in the ‘Mother-30 °C’ and ‘Pups-30 °C’ groups (Supplementary materials, Table S1). Although the RMR of litters in the ‘Mother-30 °C’ group was lower by 17.7% and 17.7% than that in the ‘Both-21 °C’ and ‘Pups-30 °C’ groups, respectively, the difference between the three groups was not statistically significant (*F*_2, 31_ = 2.55, *P* > 0.05, Table S1). A significant positive correlation was observed between GEI and RMR of females, and GEI and RMR of litters in the ‘Both-21 °C’ group, but not in the ‘Mother-30 °C’ and ‘Pups-30 °C’ groups (Figs S2B, S3B).

## Discussion

Previous work studying Swiss mice between 23 °C and 5 °C has strongly suggested that their SusEI and reproductive performance are constrained by the capacity of the mammary gland to produce milk^6^,^7^,^11^,^18^,^22^,^28^. In the present study, we were interested in whether higher temperatures than 23 °C would inhibit the SusEI and reproductive output of these mice suggesting that there is a heat dissipation limit on performance, but only at higher temperatures. Alternatively, their milk production might be independent of ambient temperature across the whole range for 30 down to 5 °C. We observed that Swiss mice lactating at 30 °C consumed 38% less food during peak lactation than those maintained at 21 °C. GEI, DEI and MEI of females at 30 °C were also significantly lower than females at 21 °C, whereas digestibility did not differ between the two temperatures. In addition, thermal conductance of the fur was not changed in females at 30 °C compared with that at 21 °C. These data suggest that during peak lactation at 30 °C Swiss mice had lower energy intake than that observed at room temperature. During lactation a large part of the energy intake is allocated to milk as the energy demands of pups for growth and activity must be met entirely by energy of milk^4^. Here we found a significant difference in milk energy output (MEO) between mice held at the two temperatures, with females at 30 °C having 43.2% lower MEO than those at 21 °C. Accordingly, litter mass and mean pup mass at 30 °C were significantly lower than that at 21 °C.

There are two alternative explanations for these data. The first possibility was that the reproductive performance might be constrained by the capacity of females to dissipate heat because the females exposed to 30 °C did not reduce the thermal conductance of the pelage and therefore must have experienced more heat stress compared with those lactating at room temperature. It has been suggested previously that the suckling unit of a female and her offspring might more easily to lead to maternal hyperthermia, as the pups surrounding the female may retard her capacity for heat loss, consequently forcing the mother to frequently discontinue suckling^13^,^29^,^30^,^31^,^32^. Frequent disruption of suckling may then influence oxytocin and prolactin production, ultimately resulting in decreased milk production^12^,^13^. We found no support for this suggestion in MF1 mice suckling at 21 °C^33^. However, consistent with this interpretation, suckling behavior in the Swiss mice observed here was affected by temperature, and hot-exposed females spent 25.0% less time on suckling their pups, but showed 31.6% more suckling bouts than those maintained at 21 °C. This suggested that hot-exposed females terminated suckling bouts more frequently than those lactating at room temperature.

The second possibility was that pup growth capacity might affect maternal energy intake and milk production^18^,^34^. Thus the lower growth of the pups may have been a direct consequence of the high ambient temperature inhibiting their growth. Moreover, pups raised at 30 °C, within the thermal neutral zone (TNZ) of this strain of mouse, probably spent less energy on temperature regulation than those raised at 21 °C. Together the lower maintenance energy and growth energy requirements would then lead to a lower requirement of milk from the female, with knock on effects for SusEI. This would be compounded by the lower demands for energy for the female herself. In fact Swiss mice females at 30 °C had 32.7% lower RMR and 35.4% lower DEE than those at 21 °C. These reductions were similar to those observed previously in MF1 mice^12^. Lower levels of RMR are potentially linked to the levels of the food that animals were consuming since increased food intake requires enlarged organs to digest, absorb and process the nutrients, and deliver nutrients and oxygen to peripheral tissues^8^,^23^,^35^,^36^,^37^,^38^,^39^,^40^. In the present study, we observed significantly lower sizes of visceral organs, including heart, liver, spleen, kidneys and digestive tracts as well as the mammary glands in Swiss mice lactating at 30 °C than those at 21 °C. However, within each group RMR was not related to food intake. Similar results were also found in MF1 mice^23^. These latter observations of mammary gland size are inconsistent with the peripheral limitation hypothesis, which suggests that the mammary gland would be at maximal size during peak lactation independent of the temperature.

Based on the results of experiment 1 we were unable to separate whether the observations were due to limitations imposed on mothers by for example heat dissipation capacity, or limits imposed on the pup growth or pup demands. In experiment 2, we manipulated litters to 6 and 9 at 30 °C. The rationale for this experiment was that if the high temperature was imposing limits on the mothers, then the performance of the mothers would be independent of how many pups they were given to raise, and the pups would differ in their ultimate weaning size. In contrast if the temperature imposed a limit on pup growth and pup maintenance requirements then we would expect the performance of the lactating females to be responsive to the litter sizes and the pup weaning sizes to be independent of litter size. The data clearly indicated that female lactation performance was the same, regardless of whether they were raising 6 or 9 pups (Table 3) and in consequence the pups from litters of 6 were weaned at significantly heavier weights than those from litters of 9. This result suggests that the system of a lactating mother and her offspring at 30 °C is controlled by limitations placed directly on the mother, rather than indirectly via limits placed on her offspring.

To further test whether the temperature imposes limitations on the mother, rather than the offspring, we performed a third experiment. To do this we exposed either only the mothers to 30 °C or only their litters to 30 °C. This type of separate exposure of mothers and their litters has been performed previously in mice using choice cages for the mother^27^. In this case we used a different protocol. We separated the mother and her pups for periods of 90 minutes, followed by 90 minutes with them together, and we repeated this throughout the day and night so that in total they were together for 12 h and separated for 12 h each day. When the mother and pups were separated we either exposed the mother to 30 °C and the litter to 21 °C, or the litter to 30 °C and the pups to 21 °C, or maintained them both at 21 °C as a control for disturbance. The rationale for this experiment was that if the system is driven by pup demands then exposing the pups alone to 30 °C would reduce their milk energy requirements, reducing demands on the females with consequent impacts on susEI and MEO. In contrast, if the system is limited by temperature impacts on the females, then we would anticipate negative effects of temperature only in the group where the females were exposed to high temperature. The results from this experiment clearly indicated that lactation performance was only reduced in the group where the female was exposed to the high temperature. Indeed growth of the pups in the group where the pups alone were exposed to 30 °C was significantly greater than the group where both pups and mother were kept at 21 °C. This was consistent with the pups saving energy at 30 °C, compared to pups held continuously at 21 °C, an energy saving they could apparently divert to greater growth. These data strongly refute the idea that the lowered performance at higher temperature is mediated by a direct effect of temperature on pup growth and lower pup maintenance demands, and support a direct negative impact of temperature on the females, consistent with the idea that heat dissipation limits maternal performance at this temperature.

Also consistent with the HDL theory was the observation that BAT *ucp1* and *cpt1b* gene expression, and UCP1 protein content, did not differ between the two temperatures, indicating that BAT thermogenesis was unchanged. BAT mass, *ucp1* mRNA and UCP1 protein levels have been previously observed to decrease during lactation in laboratory mice^5^,^24^,^41^, Brandt’s voles^42^,^43^,^44^ and ground squirrels^45^. BAT is known as the key thermogenic organ in small mammals^46^. The down regulation of the genes associated with thermogenesis and fatty acid oxidation observed in lactating animals mediate a reduction in heat production^24^,^47^. These changes are consistent with the HDL theory because they indicate animals switch off any competing sources of heat production during lactation^24^. The absence of any temperature effect in lactation on these genes is consistent with the idea that at 21 °C females at peak lactation must already have switched off the thermogenic mechanisms mediated by *ucp1* and *cpt1b*. Consequently when they were exposed to 30 °C, no additional down regulation was feasible.

### Summary

In conclusion, female Swiss mice lactating at 30 °C showed significantly less energy intake, exported less milk and weaned lighter litters than those lactating at 21 °C. RMR and DEE of females were also significantly lower at 30 °C than at 21 °C. We performed additional experiments to demonstrate that the high temperature imposed limits directly on the females, and not indirectly via effects on the pup growth and pup energy requirements. BAT *cpt1b* and *ucp1* gene expression, and UCP1 protein levels did not differ between the two temperatures. Taken together the data across the three experiments strongly suggests that at 30 °C the female Swiss mouse is limited by heat dissipation capacity, rather than capability of the mammary gland to synthesise milk. Since previous work on these mice lactating at temperatures below 23 °C indicates that in this strain the mammary gland limits performance, these data support the model proposed by Speakman and Król (2011) that different types of constraint are operational under different conditions^15^.

## Materials and Methods

All the procedures involving animals were reviewed and approved by the Animal care and use committee of the University of Wenzhou. The methods were carried out in accordance with the approved guidelines.

### Animals

Virgin female Swiss mice, 8–10 weeks old, were obtained from our breeding colony, and were group-housed and maintained in a 12:12 light: dark cycle (lights on at 08:00 h). Room temperature was kept at 21 ± 1 °C. Standard rodent chow (Beijing KeAo Feed Co., Beijing, China) and water were available *ad libitum*. Mice were housed individually in plastic cages (29 × 18 × 16 cm) with fresh sawdust bedding for 5 days before the experiment started.

**Experiment 1** was designed to examine effect of thermoneutral temperatures (30 °C) on energy intake, DEE and MEO in lactating females. Fifty females were paired with males for 11 days, after which males were removed. Forty-seven mice became pregnant and gave birth. On the day of parturition, females had their litter sizes manipulated to raise 12 offspring. Because the average litter size was less than 12 we ended up with forty two mice with litters of 12 which were randomly assigned into one of two groups: the 21 °C group (21 °C, *n* = 21), within which females continued to be maintained at 21 ± 1 °C, and the 30 °C group (30 °C, *n* = 21), females and their offspring were transferred to a hot temperature (30 ± 1 °C) throughout lactation. All pups were weaned on day 17 of lactation. Body mass and food intake of females, and litter size and mass were measured on a daily basis as described previously^11^.

### Resting metabolic rate (RMR)

RMR was measured using a computerized open-flow respirometry system (Sable Systems, USA). Air was pumped at a rate of 600–850 ml/min through a cylindrical sealed Perspex chamber, where the flow ensured adequate mixing. Gases leaving the chamber were dried (silica gel) and directed through the oxygen analyzer (FC-10A, Sable Systems) at a flow rate of 150–175 ml/min. Output from the oxygen analyzer (%) was digitized using an analogue-to-digital converter (STD-UI2, Sable systems) and recorded on a personal computer using EXPEDATA data acquisition software (Sable Systems). The sampling interval was 10 s. Measurements were made at 30 ± 0.5 °C within the thermoneutral zone^48^. On day 17 of lactation, RMR was measured for 2 hours, and was calculated from the lowest rate of oxygen consumption over 5 min using the equation: VO_2_ = FR (FiO_2_-FeO_2_)/(1- FiO_2_ × (1-RQ)), where FR is the flow rate in ml/min, FiO_2_ and FeO_2_ are the fractional concentration of O_2_ entering and leaving the chamber, respectively. RQ is a respiratory quotient of 0.85^49^,^50^,^51^.

### Behavioral observation

Suckling behavior observations were made over a day (24 h) on day 10–15 of lactation in females at 21 °C (*n* = 8) and 30 °C (*n* = 8), respectively. Observations were performed using computer-connected infrared monitors (SONY, 420 TV line) and automatically stored in a computer, which were then subjected to operator analysis. The dominant behavior was recorded as either suckling or non-suckling. If mothers did not suckle pups, whatever else they did, the behavior of females was defined as non-suckling. Suckling duration was calculated as the accumulative suckling behavior in each suckling bout.

### Energy intake and digestibility

Food was provided quantitatively, and the spillage of food mixed with bedding and feces were collected from each cage over 2 days (day 13–15 of lactation). The spillage of food and feces were sorted and separated manually after they were dried at 60 °C to constant mass. Gross energy contents of the diet and feces were measured using a Parr 1281 oxygen bomb calorimeter (Parr Instrument, Moline, IL, USA). Gross energy intake (GEI), digestive energy intake (DEI), and apparent energy assimilation efficiency (digestibility) were calculated as follows^52^,^53^:

GEI (kJ · d^−1^) = food intake (g · d^−1^) × dry matter content of the diet (%) × energy content of food (kJ · g^−1^);

DEI (kJ · d^−1^) = GEI − (dry mass of feces (g · d^−1^) × energy content of feces (kJ · g^−1^));

Digestibility (%) = DEI/GEI × 100%.

### DEE and MEO

DEE of female was measured on days 13–15 of lactation using the doubly labelled water (DLW) technique^54^. The use of which in lactation has been previously described in detail^16^. Briefly, on day 13 of lactation, females were weighed and injected intraperitoneally with approximately 0.5 g of the DLW containing enriched ^2^H and ^18^O. We weighed the syringe (to 0.1 mg) using a Sartorius balance before and immediately after the injection. Females were replaced in their cages and were allowed to free access to offspring as well as food and water. After a one-hour of isotope equilibration, we took the initial blood samples to estimate initial isotope enrichments, and 48 h later (the same time on day 15) we took final blood samples to estimate isotope elimination rates. Both initial and final blood sample collections were performed by tail tipping, and immediately sealed into two 60 μl glass capillaries using a hand-held butane torch, which were sealed again with sealing wax. DEE was calculated based on CO_2_ production as described previously^16^. MEO was calculated from the difference between metabolizable energy intake (MEI) and DEE, during which MEI was calculated from DEI × (100–3%) since urinary energy loss (UEL) was assumed to be 3% of DEI^55^.

### Body composition

Females were sacrificed by decapitation after offspring were weaned on day 17. Interscapular BAT was quickly removed and frozen immediately in liquid nitrogen and was stored at −80 °C. The whole fur including tail and limbs but excluding head was separated and stored at −20 °C for the measurements of thermal conductance. Brain, mammary gland, liver, heart, lung, spleen and kidneys were separated and weighed (to 0.1 mg). The gastrointestinal tracts including stomach, small and large intestine and caecum were also removed and weighed without content and any mesentery. These tissues were weighed again (to 0.1 mg) after they were dried at 60 °C for two weeks to constant mass.

### Thermal conductance of the fur

We wrapped the fur around a small bottle filled with water (20 ml) that contained a temperature transmitter (15.5 mm × 6.5 mm; 1.1 g) (Mini Mitter ModelG2 E-Mitter), and then attached it to the bottle with contact adhesive. An incubator (Yiheng Model LRH-250, Shanghai, China) was used to heat the bottle of water to 40 °C. After reaching to 40 °C. the bottle was immediately put into another incubator at 20 °C, and then the temperature decreasing from 40 °C to 20 °C was monitored every 15 seconds. Each data was transformed according to ln (x-20), x is temperature monitored during the temperature decreasing course. The slope calculated for these transformed data was defined as the thermal conductance^56^.

### BAT gene expression

Total RNA from Interscapular BAT was homogenized in TRIzol Reagent (Life Technologies) on ice. RNA concentration was quantified by spectrophotometry (NanoDrop Technologies, Wilmington, DE, USA). Reverse transcription of total RNA into cDNA by the M-MLV Reverse Transcripatase (Life Technologies, 28025). Real-time PCR were performed in 96-well PCR plates with Maxima SYBR Green qPCRMaster Mix (Thermo Scientific, K0252). Primer sequences used in RT-qPCR were provided below: *gapdh* (forward, 5′- ggtgaaggtcggtgtgaacg-3′, reverse, 5′-ctcgctcctggaagatggtg-3′), *cpt1b* (forward, 5′- ccagacccatacaccgacag-3′, reverse, 5′-gtctcagagcctcccgacta-3′); *pgc1α* (forward, 5-aagggccaaacagagagaga-3, reverse, 5-cgttcgacctgcgtaaagta-3); *ucp1* (forward, 5′- actgccacacctccagtcatt-3′, reverse, 5′-ctttgcctcactcaggattgg-3′). Relative expression of mRNAs was determined using the ^ΔΔ^-Ct method and normalized with *gapdh* levels.

### Western blotting

Frozen Interscapular BAT was homogenized on ice in RIPA buffer supplemented with anti-protease agents [1 mmol l^−1^ DTT, 1 mmol l^−1^ PMSF, 0.1 mmol l^−1^ EDTA, 1:1000 Protease Inhibitor Cocktails (Sigma-Aldrich)], and the homogenate was centrifuged at 4 °C at 13,000 rpm for 10 min to obtain the whole protein of the tissue. Protein concentrations were determined using the Lowry assay. Extracted protein (40 μg) was loaded and separated in a discontinuous SDS-polyacylamide gel, and then was transferred to PVDF membranes and incubated with bovine serum albumin solution overnight at 4 °C. Using antibodies against UCP1 (Abcam, ab10983) and beta-actin (ZSGB-BIO, Beijing, China) to detect protein expression level, respectively. Then the secondary antibody peroxidase-conjugated goat anti-rabbit IgG and goat anti-mouse IgG were added. Enhanced chemiluminescence (GE Healthcare, RPN2109) was used for detection. Film images results were scaned in tif file and quantified with Image software (National Institutes of Health, Bethesda, MD, USA).

**Experiment 2** was designed to test the possibility that limitation of the growth capacity of the pups was the key factor drives SusEI during peak lactation in females at hot temperature (30 °C). Twenty six females, 8–10 weeks of age, were paired with males as described above. On the day of parturition, litter size of females was artificially manipulated, allowing mothers to raise 6 pups (LS = 6 group, *n* = 12) or 9 pups (LS = 9 group, *n* = 12). The rest of pups in addition to 6 in LS = 6 group or 9 in LS = 9 group were removed. Both groups of females and their pups were maintained at 30 °C throughout lactation. Food intake and body mass of females, as well as litter size and litter mass were measured as described above. RMR and DEE of females were also measured according to the methods mentioned in experiment 1. In addition, RMR of litters were determined using the open-flow respirometry system at 30 °C as described above, but the air was pumped at a rate of 1000–1200 ml/min since the average masses of litters were 69 to 82** **g. GEI, DEI, MEI and digestibility, as well as DEE and MEO were also measured according to the method mentioned above.

**In experiment 3**, we aimed to separate the effect of elevated temperature on females from the impact of temperature on their offspring. Fifty females were paired with males at 21 ± 1 °C. On the day of parturition, all lactating females had their litter sizes manipulated to 12, and were randomly allocated to one of three groups. All groups were treated the same way for the first 11 days of lactation. After day 11 the females in the ‘Both-21 °C’ (*n* = 11) group, were removed from the cage where the pups were located (home cage) and placed in an otherwise identical cage separated from their pups for an interval of 90 min. Food and water were available in both cages. They were then reunited with their pups for 90 minutes, and this cycle continued night and day (so that they were in contact with their pups 12 h/day) until day 17 of lactation. In the ‘Both-21 °C’ group the two cages were kept at 21 °C. Two other groups, the ‘Mother-30 °C’ (*n* = 15) and ‘Pups-30 °C’ (*n* = 15) groups, were treated similarly, in that the females were also separated from their pups on a 90 minute cycle, but during the separation period the mothers were exposed to 30 °C in ‘Mother-30 °C’ group while their pups stayed at 21 °C, while the pups were exposed to 30 °C in the ‘Pups-30 °C’ group, but their mothers stayed at 21 °C. Pups from all three groups were weaned on day 17 of lactation. Litter mass and litter size were measured on a daily basis throughout lactation. Body mass and food intake were measured daily on days 1 to 10 of lactation, but on a 90 min interval during days 11 till 17. Food intake was expressed as g/d on day 1 to 17 and g/90 min on day 11 to 17 respectively. GEI, digestibility, MEI, RMR of females and pups, DEE and MEO were also measured as described under experiment 1.

### Body temperature (T_b_)

Encapsulated thermo-sensitive passive transponders (diameter 2 mm and length 14 mm, Destron Fearing, South St Paul, USA) were able to measure temperature between 25 and 40 °C according to the manufacturer’s specifications. We implanted transponders subcutaneously in the dorsolateral hip region of all females in three groups and measured their T_b_ daily on day 1 till day 17 of lactation. T_b_ measurements were performed at 16:00 once a day using a Pocket Reader (Destron Fearing, South St Paul, USA).

### Statistics

Data were analyzed using SPSS 13.0 statistical software. Changes in body mass and food intake of females as well as litter size, litter mass and mean pup mass over the course of lactation period were examined using repeated one way-ANOVA. In experiment 1, group difference between 21 °C and 30 °C in food intake, body mass, suckling behavior, thermal conductance, energy intake, DEE and MEO, and litter size, litter mass, mean pup mass were examined using independent *t* tests. In experiment 2, difference in food intake, body mass, litter size and mass, and BAT genes expression between LS = 6 and LS = 9 groups were also analyzed using independent *t* tests. Effects of temperature on RMR and body composition of females were analyzed using ANCOVA with body mass or carcass mass as a covariate where appropriate. In experiment 3, effects of temperatures on food intake, body mass, GEI, DEI, MEI, DEE and MEO, and litter size, litter mass and mean pup mass were examined using one way-ANOVA or ANCOVA, with body mass as a covariate, followed by Tukey’s HSD post hoc tests where required. Pearson’s correlation was performed to examine correlations between any two parameters presented in the text. Data were reported as means ± s.e.m. Statistical significance was determined at *P* <  0.05.

## Additional Information

**How to cite this article**: Zhao, Z.-J. *et al*. Limits to sustained energy intake XXV: milk energy output and thermogenesis in Swiss mice lactating at thermoneutrality. *Sci. Rep.* **6**, 31626; doi: 10.1038/srep31626 (2016).

## Supplementary Material

Supplementary Information

## Figures and Tables

**Table 1:** Energy intake, daily energy expenditure (DEE) and milk energy output (MEO) in Swiss mice lactating at 21 and 30°C.

	21 °C	30 °C	P
GEI (kJ/d)	377.9 ± 5.8	225.4 ± 6.5	**
DEI (kJ/d)	285.2 ± 4.7	170.9 ± 5.2	**
MEI (kJ/d)	276.7 ± 4.6	165.7 ± 5.0	**
Digestibility (%)	75.5 ± 0.4	75.8 ± 0.5	ns
DEE (kJ/d)	141.8 ± 4.6	91.6 ± 2.8	**
MEO (kJ/d)	137.0 ± 5.3	77.8 ± 4.9	**

GEI, gross energy intake; DEI, digestive energy intake, MEI, metabolizable energy intake; Data are means ± s.e.m. **Significant difference between 21 and 30 °C groups (*P* < 0.01). ns, non-significant difference (*P* > 0.05).

**Table 2:** Body composition in Swiss mice lactating at 21 and 30°C.

	21 °C	30 °C	P
Body mass (g)	51.3 ± 1.0	40.4 ± 0.9	**
Wet mass (g)			
Carcass	18.4 ± 0.3	17.3 ± 0.5	*
Brain	0.395 ± 0.011	0.387 ± 0.010	ns
Mammary glands	4.799 ± 0.218	3.032 ± 0.117	**
Liver	3.689 ± 0.120	2.651 ± 0.067	**
Heart	0.306 ± 0.010	0.226 ± 0.007	**
Lung	0.341 ± 0.025	0.263 ± 0.016	ns
Spleen	0.217 ± 0.016	0.135 ± 0.015	**
Kidneys	0.584 ± 0.013	0.455 ± 0.013	**
Stomach	0.432 ± 0.016	0.342 ± 0.016	**
Small intestine	2.250 ± 0.126	1.356 ± 0.104	**
Large intestine	0.860 ± 0.031	0.543 ± 0.021	**
Caecum	0.386 ± 0.016	0.236 ± 0.017	**
Dry mass (g)			
Carcass	5.4 ± 0.1	5.1 ± 0.1	*
Brain	0.093 ± 0.003	0.095 ± 0.005	ns
Mammary glands	1.321 ± 0.080	0.791 ± 0.032	**
Liver	1.034 ± 0.033	0.736 ± 0.020	**
Heart	0.074 ± 0.003	0.054 ± 0.002	**
Lung	0.074 ± 0.005	0.058 ± 0.003	ns
Spleen	0.052 ± 0.004	0.033 ± 0.004	*
Kidneys	0.142 ± 0.004	0.108 ± 0.003	**
Stomach	0.106 ± 0.004	0.082 ± 0.003	**
Small intestine	0.521 ± 0.023	0.295 ± 0.020	**
Large intestine	0.177 ± 0.006	0.114 ± ± 0.004	**
Caecum	0.076 ± 0.003	0.047 ± ± 0.003	**

*Significant difference between 21 and 30 °C groups (*P* < 0.05), **P < 0.01; ns, no significant difference between the two groups (*P* > 0.05).

**Table 3:** Energy intake, RMR and MEO in Swiss mice raising 9 or 6 pups at hot temperature.

	LS = 9	LS = 6	P
RMR (mlO_2_/h)			
Females	86.8 ± 10.1	72.1 ± 4.8	ns
Litters	149.5 ± 11.9	120.8 ± 9.0	ns
GEI (kJ/d)	218.3 ± 7.1	219.0 ± 7.2	ns
DEI (kJ/d)	174.4 ± 6.3	172.5 ± 7.8	ns
MEI (kJ/d)	169.1 ± 6.1	167.3 ± 7.6	ns
Digestibility (%)	79.8 ± 0.7	78.5 ± 1.4	ns
DEE (kJ/d)	84.1 ± 2.9	82.8 ± 1.6	ns
MEO (kJ/d)	90.3 ± 4.5	89.7 ± 7.2	ns

RMR, resting metabolic rate; GEI, gross energy intake; DEI, digestive energy intake; MEI, metabolizable energy intake; MEO, milk energy output; LS, litter size. Data are means ± s.e.m. ns, non-significant difference between the two groups (*P* > 0.05).

**Table 4:** Food intake (g/d) of females when mother was moved out and back in the cage from day 1 to 17 of lactation.

Day of lactation	Both-21 °C	Mother-30 °C	Pups-30 °C	*F*	*P*
Mother out					
11	18.8 ± 1.0	16.4 ± 0.6	19.4 ± 0.8	3.86	*
12	19.2 ± 0.9	17.0 ± 0.5	19.1 ± 0.9	3.11	0.06
13	19.8 ± 1.0	17.0 ± 0.8	18.9 ± 0.6	2.69	0.08
14	17.8 ± 0.8	16.6 ± 1.0	18.9 ± 0.8	2.47	0.10
15	20.0 ± 0.6	16.7 ± 0.9	18.6 ± 0.6	4.42	*
16	17.9 ± 0.5	17.2 ± 0.9	19.4 ± 0.7	3.53	*
17	18.1 ± 0.5	16.8 ± 0.8	18.0 ± 0.8	0.77	ns
Accumulative FI	131.6 ± 3.6	119.4 ± 3.9	130.9 ± 4.4	2.832	0.07
Mother in					
11	6.6 ± 0.4	5.5 ± 0.5	5.6 ± 0.6	1.67	ns
12	6.4 ± 0.6	4.8 ± 0.5	6.2 ± 0.6	2.17	ns
13	6.9 ± 0.4	5.4 ± 0.7	7.0 ± 0.6	2.26	ns
14	7.7 ± 0.8	5.0 ± 0.7	7.2 ± 0.7	4.77	*
15	6.7 ± 0.7	5.2 ± 0.7	8.5 ± 0.6	7.27	**
16	7.8 ± 0.5	4.3 ± 0.7	7.7 ± 0.8	7.58	**
17	6.6 ± 0.9	5.4 ± 0.7	9.9 ± 0.9	7.00	**
Accumulative FI	48.6 ± 2.3	34.6 ± 3.5	51.4 ± 3.1	8.57	**

Accumulative FI, sum of food intake between day 11 to 17. Data are means ± s.e.m. ns, non-significnt difference (*P* > 0.05); *significant difference between the three groups (*P* < 0.05), **P < 0.01.
