# Supplementary material for: Limits to sustained energy intake XXV: milk energy output and thermogenesis in Swiss mice lactating at thermoneutrality
Source: Sci Rep. 2016 Aug 24;6:31626. doi: 10.1038/srep31626 (PMC4995430; doi:10.1038/srep31626)
Supplement: Supplementary Information [file srep31626-s1.doc]

**Limits to sustained energy intake XXV: milk energy output and thermogenesis in Swiss mice lactating at thermoneutrality**

Zhi-Jun Zhao^1^*, Li Li^2^, Deng-Bao Yang^2,3^, Qing-Sheng Chi^3^, Catherine Hambly^4^, John R. Speakman^2,4^*

1. School of Life and Environmental Sciences, Wenzhou University, Wenzhou, Zhejiang 325027, China

2. State Key Laboratory of Molecular Developmental Biology, Institute of Genetics and Developmental Biology, Chinese Academy of Sciences, Beijing 100100, China

3. State Key Laboratory of Integrated Management for Pest Insects and Rodents, Institute of Zoology, Chinese Academy of Sciences, Beijing 100080, China

4. Institute of Biological and Environmental Sciences, University of Aberdeen, Aberdeen, Scotland, UK

^*^Author for correspondence

Zhi-Jun Zhao, Ph.D.

Tel: +86-577-86689079

Fax: +86-577-86689257

Email: [zhao73@foxmail.com](mailto:zhao73@foxmail.com)

and

John R Speakman

Tel: +44 (1224) 272879

Fax: +44 (1224) 272396

E-mail: [J.Speakman@abdn.ac.uk](mailto:J.Speakman@abdn.ac.uk)

A

Gene expression fold-change

*cpt1b* *pgc1a*   *ucp1*

B

Protein content (relative unit)

UCP1

Figure S1

Zhao ZJ et al.

Figure S1. Gene expression of BAT *cpt1b*, *pgc1a* and *ucp1* (A), as well as UCP1 protein content (B) in Swiss mice at 21 °C and 30 °C. Data are means ± s.e.m.

*r*=0.82, *P*<0.05

*r*=0.29, *P*>0.05

*r*=0.14, *P*>0.05

A

GEI (kJ/d)

Body mass (g)

*r*=0.90, *P*<0.01

*r*=0.47, *P*>0.05

*r*=0.19, *P*>0.05

B

GEI (kJ/d)

RMR of females (mlO2/h)

*r*=0.98, *P*<0.01

*r*=0.65, *P*<0.05

*r*=0.60, *P*<0.05

C

GEI (kJ/d)

DEE (kJ/d)

*r*=0.98, *P*<0.01

*r*=0.89, *P*<0.01

*r*=0.86, *P*<0.01

D

GEI (kJ/d)

MEO (kJ/d)

Figure S2

Zhao ZJ et al.

Fig. S2. Correlations between gross energy intake (GEI) and body mass (A), RMR of females (B), daily energy intake (DEE, C) and milk energy output (D) in Swiss mice during peak lactation. Data are plotted.

*r*=0.86, *P*<0.01

*r*=0.60, *P*<0.05

*r*=0.59, *P*<0.05

GEI (kJ/d)

Litter mass (g)

A

*r*=0.76, *P*<0.05

*r*=0.56, *P*=0.06

*r*=0.27, *P*>0.05

GEI (kJ/d)

RMR of litters (mlO_2_/h)

B

Figure S3

Zhao ZJ et al.

Fig. S3. Correlations between gross energy intake (GEI) and litter mass (A) and RMR of litters (B) in Swiss mice during peak lactation

| Table S1 Resting metabolic rate (RMR) of females and litters in Swiss mice during peak lactation | | | | | |
| --- | --- | --- | --- | --- | --- |
| Day of lactation | Both  -21°C | Mother  -30°C | Pups  -30°C | *F* | *P* |
| Body mass (g) | 47.7±1.8^ab^ | 45.6±0.8^b^ | 50.2±1.0^a^ | 4.77 | * |
| RMR (mlO_2_/h) | 206.4±11.9 | 183.6±9.0 | 184.1±10.3 | 1.30 | ns |
| Litter mass (g) | 97.3±3.6^b^ | 82.3±3.0^c^ | 117.3±3.7^a^ | 27.89 | ** |
| RMR of litters |  |  |  |  |  |
| (mlO_2_/h) | 258.2±19.3 | 212.5±14.5 | 258.1±18.4 | 2.55 | 0.09 |
| Data are means ± s.e.m. ns, non-significant difference (*P*>0.05); *, significant effect of temperatures (*P*<0.05), **, *P*<0.01. Different letters on the same row indicate significant difference between the three groups (*P*<0.05). | | | | | |
